# Oxidation of Mesalamine under Phenoloxidase- or Peroxidase-like Enzyme Catalysis

**DOI:** 10.3390/molecules28248105

**Published:** 2023-12-15

**Authors:** Rimaz El Zein, Pompilia Ispas-Szabo, Maziar Jafari, Mohamed Siaj, Mircea Alexandru Mateescu

**Affiliations:** Department of Chemistry and Center CERMO-FC, Université du Québec à Montréal, Downtown Branch, P.O. Box 8888, Montréal, QC H3C 3P8, Canada; el_zein.rimaz@courrier.uqam.ca (R.E.Z.); ispas-szabo.pompilia@uqam.ca (P.I.-S.); jafari.maziar@courrier.uqam.ca (M.J.); siaj.mohamed@uqam.ca (M.S.)

**Keywords:** 5-ASA (5-aminosalicylic acid), acetaminophen, ceruloplasmin, hemoglobin, hydrogen peroxide, hydroquinone, inflammatory bowel diseases, laccase, mesalamine, peroxidase

## Abstract

Mesalamine, also called 5-ASA (5-aminosalicylic acid), is a largely used anti-inflammatory agent and is a main choice to treat Ulcerative Colitis. This report is aimed to investigate enzymatic processes involved in the oxidation of mesalamine to better understand some of its side-effects. Oxidation with oxygen (catalyzed by ceruloplasmin) or with hydrogen peroxide (catalyzed by peroxidase or hemoglobin) showed that these oxidases, despite their different mechanisms of oxidation, could recognize mesalamine as a substrate and trigger its oxidation to a corresponding quinone-imine. These enzymes were chosen because they may recognize hydroquinone (a *p*-diphenol) as substrate and oxidize it to *p*-benzoquinone and that mesalamine, as a *p*-aminophenol, presents some similarities with hydroquinone. The UV-Vis kinetics, FTIR and ^1^H NMR supported the hypothesis of oxidizing mesalamine. Furthermore, mass spectrometry suggested the quinone–imine as reaction product. Without enzymes, the oxidation process was very slow (days and weeks), but it was markedly accelerated with the oxidases, particularly with peroxidase. Cyclic voltammetry supported the hypothesis of the oxidative process and allowed a ranking of susceptibility to oxidizing mesalamine in comparison with other oxidizable drug molecules with related structures. The susceptibility to oxidation was higher for mesalamine, in comparison with Tylenol (acetaminophen) and with aspirin (salicylic acid).

## 1. Introduction

A variety of chronic inflammatory dysfunctions, such as inflammatory bowel diseases (IBD), including Crohn’s disease (CD) and ulcerative colitis (UC), may affect the whole gastrointestinal tract or, specifically, the colorectal segment [1]. Mesalamine [2,3], also called 5-ASA (5-aminosalicylic acid), has been used for decades as a long-term treatment for these pathologies. The biochemical mechanisms of these diseases are not completely elucidated, triggering certain difficulties in designing the optimal formulation and treatment to be prescribed [3,4,5]. Mesalamine is a major anti-inflammatory agent that is recognized as the first line of treatment for mild-to-moderate active UC, and also as a maintenance drug for a long-term treatment to prevent symptoms from recurring [6]. However, there is a need of repetitive doses of the drug for optimal results [7]. In fact, there are some areas of uncertainty regarding the use of 5-ASA related to optimal dose for adequate response, to product formulation, to preservation of patient adherence and, more recently, to the role of mesalamine in cancer chemoprophylaxis [8].

Mesalamine is generally much better tolerated than its congeners; however, various cases of fever and rashes have been reported as a post-treatment side effect in addition to serious cases such as pancreatitis [8]. These side effects are probably not due to mesalamine itself but to its oxidized form, especially since the *p*-aminophenol functional groups present in this anti-inflammatory agent, are easily oxidized in the presence of enzymes such as ceruloplasmin (CP) and other oxidative agents in the organism [9].

It was also hypothesized that certain beneficial effects of mesalamine may be related to its oxidized form. When oxidized by HOCl (a strong oxidant generated in gut inflammation) 5-ASA can activate nuclear factor erythroid 2—related factor 2 (Nrf2) while native 5-ASA is not effective [10,11]. The Nfr2 is a transcription factor involved in regulation of the cellular defense against oxidative and toxic injuries. Its role is to render cells resistant to inflammation and chemical carcinogens [11]. Activation of Nrf2 by oxidized form of 5-ASA can lead to induction of hemeoxygenase (HO), an anti-inflammatory enzyme (EC 1.14.99.3) as a pathway involved in the anti-inflammatory effect of 5-ASA [10].

Acetaminophen (paracetamol, Tylenol), a very common analgesic and antipyretic, and mesalamine, an anti-inflammatory from the aminosalicylates’ class, are both drugs belonging to the aminophenol family. The overdose of Tylenol, easily oxidized, can cause common major adverse effects such as severe hepatotoxicity [12]. It is well documented that the oxidation of acetaminophen by CYP enzymes yields a reactive metabolite NAPQI (N-acetyl-p-benzoquinone imine), which is primarily responsible for acetaminophen-induced hepatotoxicity [13]. This fate of acetaminophen and the limited structural commonality between mesalamine and acetaminophen motivated us to initiate this investigation.

From these perspectives, it was interesting to investigate the oxidation of mesalamine by dissolved oxygen or by hydrogen peroxide in the presence of oxidases. These oxidative processes may generate a better understanding of mesalamine action and of some related beneficial or harmful side effects. The catalytical or none catalytical oxidative processes may generate oxidized forms of mesalamine. The investigation was first conducted with vegetal enzymes as laccase and peroxidase known for their capacity to oxidize *para*-diphenols or *para*-aminophenols, mimicking the activity of various animal enzymes. Then, the study was continued with the animal enzyme as ceruloplasmin (presenting an action specificity similar to that of laccase) and with hemoglobin (exerting an enzyme activity like that of the peroxidase).

Laccase (*p*-diphenoloxidase) is a multi-copper oxidoreductase: an enzyme involved in the catalytic oxidation of polyphenolic compounds [14,15], forming phenoxy radicals [16] with final generation of quinones that may induce color changes with the modification of UV-Vis spectra [17]. Laccase and ceruloplasmin are both oxidoreductases acting on molecular oxygen (O_2_) as an acceptor of electrons and on diphenols (*p*-quinols), aminophenols and phenylenediamine-related substances, as donors. In addition to its action on aminophenols, Ceruloplasmin, as an animal enzyme [18], oxidizes aromatic biogenic amines and Fe(II) to Fe(III), also known as ferroxidase.

Among the reactions catalyzed by oxidases acting on oxygen as acceptor of electrons, the oxidation of hydroquinone (HQ) to *p*-benzoquinone (*p*-BQ) with CP was investigated by Peisach and Levine [19] and with laccase by Mateescu et al. [20].

Peroxidases are enzymes that can catalyze the oxidation of a substrate by hydrogen peroxide [21]. The majority of peroxidases are ferric heme proteins, and they are present in virtually all living species [22].

Hemoglobin (Hb) is a hemoprotein mainly acting as oxygen carrier. It is also known to act in the presence of H_2_O_2_, as a peroxidase [23]. In red blood cells, this Hb activity is controlled by the reducing environment. Extracellular, free Hb has a high potential to become a peroxidase. This potential is further increased by inflammatory cells generating superoxide, easily converted into H_2_O_2_ as a substrate of the Hb peroxidase activity, possibly acting on mesalamine [24].

The rationale of the choice of these enzymes was that all of them are supposedly involved in the oxidation of substrates of hydroquinone type, which is a *p*-diphenol, and that mesalamine, as a *p*-aminophenol, presents some similarities with HQ.

Taking into consideration the similarity of mesalamine with *p*-diphenols (HQ) acting as substrates for *p*-diphenoloxidases (i.e., laccase) and for ceruloplasmin and with aromatic biogenic amines as substrates for ceruloplasmin, an involvement of these two enzymes in mesalamine oxidation was hypothesized. Furthermore, considering the release of hydrogen peroxide in various physiological and pathological conditions (e.g., activation of macrophage and of neutrophil cells), an oxidation of MS with H_2_O_2_ under the catalytic action of peroxidase and of hemoglobin was also hypothesized.

This research was focused on the enzymatic oxidation processes of mesalamine with enzymes that can possibly oxidize it in vivo.

## 2. Results and Discussion

The spectrum of native MS presents a major band, abruptly decreasing over the range 200 nm–260 nm, and another band with a maximum at 330 nm.

### 2.1. Effect of Time in the Mesalamine Oxidation

**Figure 1:**
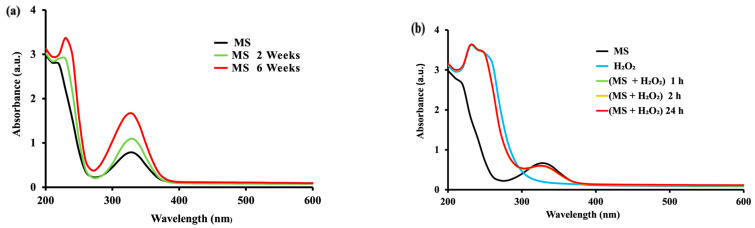
UV–Vis (200–600 nm) spectra of MS recorded at various times of (**a**) MS (0.55 mM) alone at 24 h, 2, and 6 weeks and (**b**) MS (0.55 mM) in presence of H_2_O_2_ (0.05%) up to 24 h in PBS (50 mM). The spectra are representative of at least *n* = 3 scans for each experimental condition (all run at room temperature).

In the absence of oxidative enzymes, mesalamine did not show any change in the first 24 h, indicating no oxidation with dissolved oxygen only (Figure 1a). However, such a spontaneous oxidation process occurs at longer exposure times (2 to 6 weeks), showing a slight modification in the UV-Vis spectrum of MS (Figure 1a) with a new band at about 230 nm and an increase in the band at 330 nm. With the time, these bands were slowly but continuously increased for 6 weeks (Figure 1a), suggesting a low process of oxidation in enzyme-free solutions at room temperature.

Similarly, no oxidation of MS was observed with H_2_O_2_ after 1 h, 2 h and even at 24 h (Figure 1b) in the absence of peroxidases, with superposed spectra suggesting a certain stability of mesalamine for at least 24 h in the absence of oxidative enzymes.

**Figure 2:**
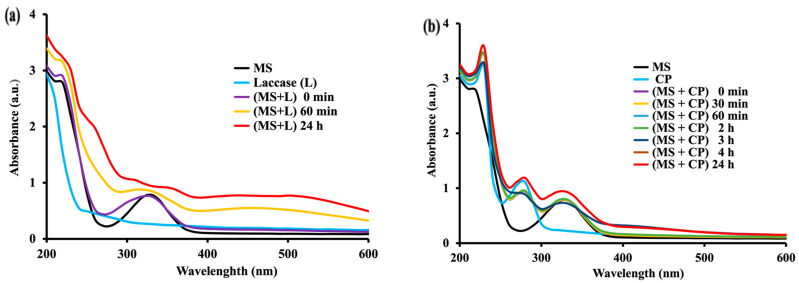
UV–Vis (200–600 nm) of MS (0.55 mM) and its oxidation product in PBS (50 mM) at room temperature in presence of (**a**) Laccase (0.030 U); (**b**) Ceruloplasmin (0.030 U) prepared to present the same number of enzyme units as laccase. Spectra are representative of at least *n* = 3 scans for each experimental condition.

A slightly lower absorbency for MS in the presence of H_2_O_2_ at 1 h–24 h than for MS alone may be explained by a partial decomposition of H_2_O_2_ that can occur above pH 5, and considering that the measurements (Figure 2b) have been carried-out at pH 7.0 (in PBS), this can explain the partial decomposition of hydrogen peroxide generating the decrease in absorbency.

Since the oxidation changes found for MS in the absence of oxidases were negligible for times of hours or days (with only some effects observed after 2 and 6 weeks), it was of interest to investigate MS oxidation in the presence of some oxidases potentially involved in the oxidation of *p*-diphenols and of *p*-aminophenols.

### 2.2. Oxidation of Mesalamine Using Vegetal and Animal Enzymes

Two types of oxidases have been investigated as catalysts for the oxidation of mesalamine: (i) laccase and CP (both acting with the molecular O_2_) and (ii) peroxidase and Hb (both acting with H_2_O_2_).

#### 2.2.1. Oxidation of MS with Molecular Oxygen Catalyzed by Laccase and by Ceruloplasmin

To have an answer to the question whether phenoloxidases can accelerate the oxidation of mesalamine, we first investigated the laccase as a copper–oxidase with catalytic properties similar, but not identical, to those of ceruloplasmin.

Both laccase and CP are enzymes able to oxidize *p*-diphenols or related aromatic phenolic substrates (SH_2_, e.g., HQ) to corresponding quinones (Q), e.g., *para*-benzoquinone *(p*-BQ) with the dissolved oxygen, as shown on the scheme:

E SH_2_ + ½ O_2_ ---------> Q + H_2_O

where E = Enzyme as laccase or as ceruloplasmin.

SH_2_ = Substrate such as HQ or as MS.

Q = Oxidized substrate such as *p*-BQ or supposedly oxidized quinone-imine of MS.

The UV-Vis spectra showed that laccase (vegetal *p*-diphenol oxidase) strongly increased the oxidation rate of MS (Figure 2a) with increasing bands in UV (with a new maximal absorbency at 230 nm over the region of 230 nm–280 nm) and with slightly increasing in intensity broader flat bands in visible region (380 nm–600 nm). These preliminary results clearly showed that an oxidation of MS occurs and that the process is accelerated by the laccase copper–oxidase.

This aspect is of interest, showing that MS is susceptible to oxidation by phenoloxidases and related enzymes. Laccases, as blue-copper oxidase-type enzymes, are endowed with 4 copper atoms/molecule. They are able to catalyze the oxidation of phenolic compounds with molecular oxygen as acceptor [25]. This suggests that mesalamine—a molecule with *p*-amino phenolic groups—could also be easily oxidized by other multi-copper enzymes such the blood circulatory ceruloplasmin.

CP is a blue-copper enzyme with six copper atoms/molecule. It is an ubiquitarian mammalian circulatory enzyme present at regular levels in physiological conditions and at high values in inflammatory conditions. It was of interest to investigate the effect of CP on MS, particularly considering that amounts of MS orally administered and, in part, even from enema products [26], would be adsorbed [27]. Thus, the probability of contact between MS and circulatory CP is high. Furthermore, MS, as an anti-inflammatory agent, is given in inflammatory conditions (when the CP level is high). Therefore, it appeared worthwhile to investigate the double risk of interactions between CP and MS (higher dosage of MS and increased level of CP). Indeed, our investigation found that CP was able to catalyze the oxidation of MS (Figure 2b) in a similar way to that catalyzed by the laccase. In both cases, the mesalamine oxidation with the mentioned oxidases was much faster than the oxidation of mesalamine alone (in enzyme-free conditions). As in the case of laccase, the increase in band intensity at 330 nm for MS in the presence of CP was higher (A_max_ = 0.9381) in 24 h (Figure 2b) compared with the increase in band intensity (A_max_ = 0.7864) for MS alone (Figure 1a).

In order to evaluate the potential impact of the various enzymes on the MS oxidation in function of their specificities of action, the oxidation kinetics have been conducted with the same MS substrate concentration (0.55 mM) for the whole investigation. The two oxidases, laccase and ceruloplasmin, acting with molecular (O_2_) dissolved oxygen as acceptor, have been prepared to present the same enzyme activity in terms of Enzyme Units (U) with HQ as a substrate where one U equaled the amounts of an enzyme needed for the oxidation of one μmole of substrate/min [20]. The choice of HQ was because it is a common substrate for all four enzymes used, and because its structure (*para*-diphenol) has some similarities with that of the MS (*para*-aminophenol compound). Therefore, HQ was a good model substrate to evaluate the relative activities of all the investigated oxidases.

Practically, the oxidation studies with both laccase and CP oxidases have been conducted with the same number of units: 0.030 U for each of the two enzymes. The relative susceptibility to oxidation of MS under the catalysis with the two enzymes was expressed as the rate of absorbency increase (ΔA/time, measured at wavelengths where there are maximal variations of spectral pattern) under catalysis of the same number of enzyme units.

The results indicated an oxidation of MS under the laccase (0.030 U) catalysis with the rates: ΔA_230nm_/min = 0.00380 Au/min and ΔA_330nm_/min = 0.00291 Au/min. The rates of oxidation of MS catalyzed by CP (0.030 U) have been evaluated as ΔA_230nm_/min = 0.00353 Au/min and ΔA_330nm_/min = 0.00244 Au/min (Table S1, Supplementary Data). These results suggest a slightly faster oxidation of MS catalyzed by laccase than that catalyzed by CP, and this can be explained by differences in terms of specificity for substrate of the two enzymes.

#### 2.2.2. Oxidation of MS with Hydrogen Peroxide Catalyzed by Peroxidase and Hemoglobin

Since MS is administered to treat inflammatory dysfunctions and considering that in these conditions there is an enhanced production of hydrogen peroxide (particularly by activated macrophage and neutrophil cells), it was of interest to evaluate the effect of H_2_O_2_ on MS in the absence (Figure 1b) and in the presence of peroxidase or of related peroxidase-like enzymes (such as hemoglobin) that could oxidize MS in circulation or in tissues (intracellularly, in peroxisomes).

Peroxidases are enzymes able to oxidize various substrates, including *p*-diphenols or related aromatic phenolic substrates (SH_2_/e.g., HQ) to corresponding quinones (Q/e.g., *p*-BQ) with hydrogen peroxide, following the scheme:

E SH_2_ + H_2_O_2_ ----> Q + 2H_2_O

where E = Enzyme as peroxidase or peroxidase-like proteins, such as Hb,

SH_2_ = Substrate such as HQ, MS

Q = Oxidized substrate such as *p*-BQ or supposedly oxidized quinone-imine of MS.

As model peroxidases, we have retained the horseradish peroxidase (HRP) and hemoglobin (Hb)—a heminic protein able to exert a peroxidase-like enzyme activity, particularly after erythrocyte lysis. Both horseradish peroxidase and peroxidase-like Hb are hereto called “peroxidases”. As for laccase and CP, the amounts of HRP and of Hb have been chosen such as to exert equal enzyme activities in terms of enzyme units, allowing thus a comparison between the capacities of the two investigated enzymes to induce the MS oxidation. Since the oxidative conditions under the peroxidase or Hb catalysis occurred in the presence of H_2_O_2_ (but by different mechanisms), the oxidation processes have been conducted with much lower volumes/amounts of peroxidases (corresponding to catalytic amounts of 1.20 mU) for both HRP and Hb.

To better understand the role of specificities of action of peroxidases in the differences, in terms of the MS oxidation rates, the oxidation studies with horseradish peroxidase (HRP) and with Hb were conducted in similar conditions, as well as concentrations of MS (0.55 mM in PBS 50 mM) for laccase and CP but in the presence of H_2_O_2_ (0.05%), and data were recorded at various times at room temperature. Again, to compare the effects of the used HRP and Hb, their activities have been determined with the hydroquinone common substrate [19] and they have been prepared to present the same number of enzyme units (1.20 mU) for both peroxidase and Hb.

**Figure 3:**
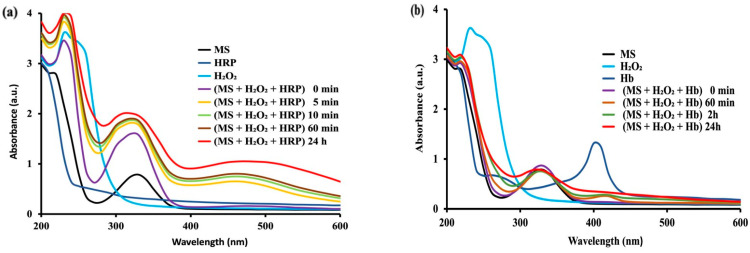
The UV–Vis spectra (200–600 nm) of MS (0.55 mM in PBS 50 mM) recorded at various times at room temperature with H_2_O_2_ 0.05% in the presence of (**a**) Horseradish peroxidase (1.20 mU), or (**b**) Hemoglobin (1.20 mU), in 50 mM PBS. Spectra are representative of at least *n* = 3 scans for each experimental condition.

Figure 3 shows that peroxidase (HRP) and Hb exerted a marked oxidative effect on MS. It was first found that peroxidase can trigger a rapid oxidation of MS with H_2_O_2_ (Figure 3a), faster than that induced by Hb (Figure 3b). The action of peroxidase (Figure 3a) consisted in time-dependent major increases in bands at 230–280 nm and at 330 nm, and also in new broad bands of moderate intensity in the large region of 400–600 nm. This effect of HRP was much faster (5–60 min) than for any other tested enzyme-catalyzed systems (with effect in tens of minutes). As far as Hb is concerned, the oxidation of MS was fast enough (even in the absence of Haptoglobin as enhancer of peroxidase-like activity of Hb), but slower than that generated by HRP.

The MS oxidation rates have been expressed, as for laccase and for CP, in terms of absorbance units as DA/min at characteristic wavelengths with maximal absorbency of the MS oxidation products (230 nm, 330 nm and 460 nm). The results indicated, for peroxidase (1.20 mU), the most rapid oxidation of MS as follows: ΔA_230nm_/min = 0.0476 Au/min, ΔA_330nm_/min = 0.0466 Au/min and ΔA_460nm_/min = 0.0994 Au/min. The MS was also oxidized with H_2_O_2_ under the catalysis of Hb (peroxidase-like activity), but the oxidation speed was definitely lower: ΔA_230nm_/min = 0.0170 Au/min, ΔA_330nm_/min = 0.0031 Au/min and ΔA_460nm_/min = 0.00064 Au/min (Table S1, Supplementary Data) than that found for peroxidase. In fact, the fastest oxidation of MS was found with H_2_O_2_, catalyzed by peroxidase.

Notably, the oxidation patterns, as reflected by the UV-Vis spectra, differed (Figure 2 and Figure 3) when the process was catalyzed by different types of oxidases. For instance, for MS oxidation with molecular O_2_, the spectra obtained under CP catalysis (Figure 2b) are more complex (particularly in the range 250–280 nm) than those obtained with laccase (Figure 2a). Furthermore, the MS oxidation with H_2_O_2_ under peroxidase catalysis is associated with a marked increase of absorbency in the range 400–600 nm (Figure 3a). This broad absorption is clearly less pronounced under Hb catalysis and almost absent in case of laccase or CP. Interestingly, the observation of a minor but well-defined band with maximal absorption at 414 nm (Soret band) [28], was ascribed to the heminic group of peroxidase (Figure 3a), and a stronger one from Hb (Figure 3b). However, the broad bands found that, for peroxidase-catalyzed MS oxidation covering the range 400–600 nm, are not related to this Soret band (which is sharp). These different patterns may suggest more than one oxidation product of MS, but a major one with a structure of quinone imine (Figure 9).

The conclusion of this section of the investigation was that, although a spontaneous oxidation (Figure 1) is very slow (days and weeks), showing a certain stability of MS, when an oxidase is present, the oxidation process can be fast (minutes), leading to different products. In order to have more information on MS oxidation, an electrochemical study was conducted by cyclic voltammetry. This investigation was also aimed to evaluate the susceptibility of oxidation of MS, in comparison with similar drugs (i.e., Tylenol, known for its possible oxidation in several hours or a few days after administration).

### 2.3. Evaluation of Susceptibility to Oxidation of Mesalamine by Cyclic Voltametry

**Figure 4:**
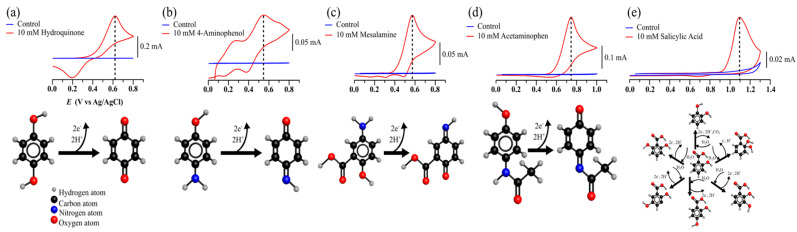
Anodic cyclic voltammetry scans of hydroquinone: 0.62 V (vs. Ag/AgCl) (**a**) of 4-aminophenol: 0.55 V (vs. Ag/AgCl) (**b**), of mesalamine: 0.58 V (vs. Ag/AgCl) (**c**), of acetaminophen: 0.75 V (vs. Ag/AgCl) (**d**), and of salicylic acid: 1.1 V (vs. Ag/AgCl) (**e**), in aqueous solution. Molecular models schematize the possible oxidation pathways.

Both acetaminophen and MS belong the aminophenol, and, consequently, it was interesting to evaluate MS’ susceptibility to oxidation compared to that of structurally related current pharmaceutics such as acetaminophen and salicylic acid. Their susceptibilities to oxidation have been compared to those of simpler compounds, such as hydroquinone and *p*-aminophenols, by cyclic voltammetry, and were ranked in terms of proneness to oxidation. All compounds showed a strong oxidation peak in the presence of an oxidative current when compared to the control solution (Figure 4). The oxidation peaks of mesalamine, acetaminophen and salicylic acid were herein found to be 0.58, 0.75 and 1.1 V (vs. Ag/AgCl), respectively (Figure 4), and in good agreement with previous reports [29,30,31,32,33,34,35].

The oxidation potentials enabled ordering the compounds based on their ease of oxidation: mesalamine (most prone to oxidation) > acetaminophen > salicylic acid (least prone to oxidation). Moreover, the anodic voltametric curves of mesalamine (Figure 4) were close to those of hydroquinone and 4-aminophenol, and this is explained by the similarity in their structures and in their oxidation mechanisms [33,34,35].

### 2.4. FTIR, ^1^H NMR and HRMS of the Oxidized Mesalamine

**Figure 5:**
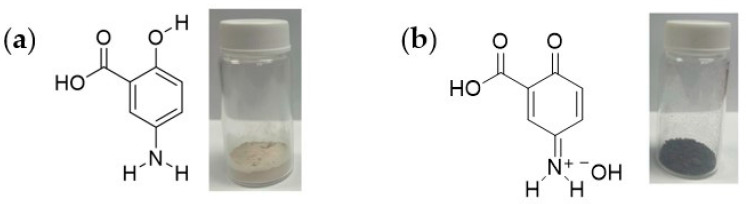
Canonical chemical structures and appearance of (**a**) mesalamine powder before oxidation processing and (**b**) of lyophilized mesalamine after non catalytical, spontaneous oxidation in aqueous solution for 6 weeks at room temperature.

The slow oxidation of mesalamine without catalysis was found after 2 and 6 weeks (Figure 1a), with a final color change from white (Figure 5a) to black (Figure 5b), similarly with the slow oxidation of acetaminophen.

**Figure 6:**
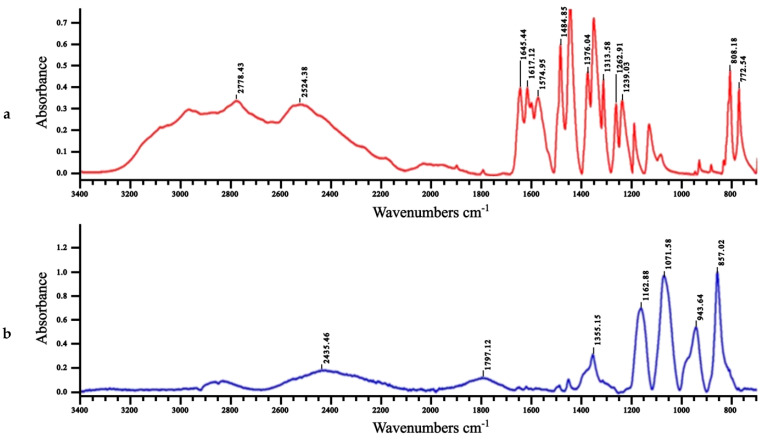
Fourier-transform infrared (FTIR) spectra of (**a**) mesalamine and of (**b**) oxidized mesalamine.

The color change of mesalamine after 6 weeks (Figure 5) was considered as a modification of its structure, related with the spontaneous oxidation process. This was supported by Fourier-transform infrared (FTIR) spectra (Figure 6) showing the disappearance of the large band around 3200 cm^−1^ ascribed to hydroxyl phenolic groups [36], and the appearance of a new band at around 1700 cm^−1^, characteristic of the novel ketone group of the supposed quinone–imine structure [37]. Also, the strong band 1342–1266, ascribed to the –N stretching aromatic amine, is not present in the oxidized MS. For mesalamine, the O-H stretch from the carboxylic acid group overlapped with N-H stretching vibrations of the amine group in the region of 2500-3500 cm^−1^. The -NH_2_ group vibration changed from a few broad peaks of MS to one broad peak near 2500 cm^−1^ in the oxidation product of MS. The N-H vibrations are influenced by hydrogen bonding, which cause the N-H stretching frequency to shift to lower wavenumbers (red shift) and broaden the absorption peak.

**Figure 7:**
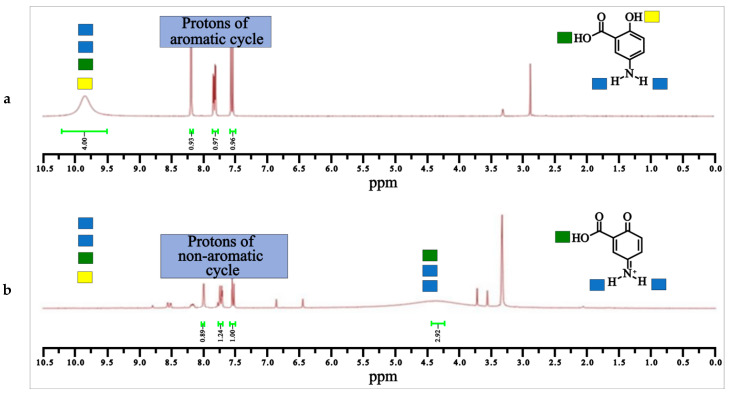
^1^H NMR of (**a**) MS and of (**b**) oxidized MS form (**b**), (300 MHz, DMSO).

The oxidation of mesalamine was also supported by ^1^H NMR spectrometry (Figure 7).

Interestingly, for mesalamine, the OH and NH_2_ peaks normally found at 5–6 ppm, appeared shifted (downfield), probably due to the intramolecular hydrogen bonding of MS between the NH_2_ group and the OH group (Figure 7a). This interaction caused significant deshielding of these protons, resulting in a downfield shift. Moreover, intermolecular hydrogen bonding with solvent molecules and other mesalamine molecules further deshielded the protons and shifted the peaks more downfield (Figure 7a). The aromatic ring, to which both the OH and NH_2_ groups are attached, have an electron-withdrawing effect, especially since the ring is substituted with the electron-withdrawing carboxylic group. This additionally contributes to the deshielding of the protons and causes a downfield shift (Figure 7a). The lone pair of electrons on the nitrogen of the NH_2_ group participates in resonance with the aromatic system. The same occurs with the lone pairs of oxygen in the OH group. These electron delocalization effects lead to an even greater deshielding of the hydrogen atoms (Figure 7a). These combined effects result in the affected protons to appear at a higher ppm value than simulated by prediction algorithms.

**Figure 8:**
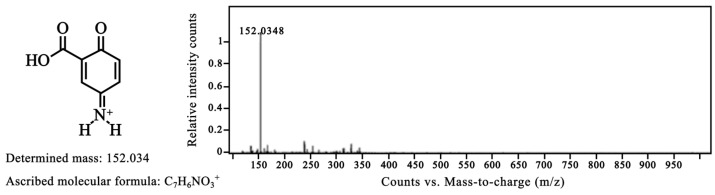
High resolution mass spectrometry (HRMS) corresponding to the oxidized mesalamine.

When mesalamine was oxidized (Figure 7b) with the loss of aromaticity in favor of a quinone form, the integrating peak for four nonaromatic protons of mesalamine (at 10 ppm) markedly shifted upfield at 4.5 ppm and integrated the three protons of the oxidized mesalamine (also related to a shielding effect). The loss of the phenolic proton is compatible with the new quinone–imine oxidation product of mesalamine (Figure 8).

**Figure 9:**
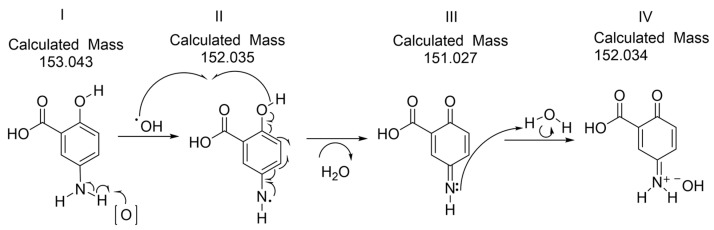
Suggested oxidation mechanism of MS in the human body. [O] stands for both hydrogen peroxide and oxygen species.

Indeed, High-Resolution Mass spectrometry analysis (Figure 8) suggested that the oxidation reaction progressed up to the form with structure IV (Figure 9) that corresponds to a mesalamine quinone–imine product having the same measured mass (152.034 g/mol) as the theoretical (calculated) molecular mass. The molecular mass of mesalamine is of 153.14 g/mol (CAS No.: 89-57-6) [3].

This supports the putative oxidative process of mesalamine in the studied system that may occur in a similar manner as in human body.

The oxidized form of MS, as supported by UV-Vis, FTIR, ^1^H NMR, cyclic voltammetry and finally by HRMS, can be hypothetically associated with the following mechanism of the reaction (Figure 9) and with the canonical chemical structures:

It is possible to hypothesize that, considering the similarity between mesalamine and acetaminophen (both belong the aminophenol class), the microsomal Cytochrome P450 system [13] involved in acetaminophen oxidation would also be involved in the oxidation of mesalamine. This hypothesis is supported by the fact that peroxidase is the enzyme triggering the fast oxidation of mesalamine and that peroxidases are among the major components of the is microsomal CYP enzyme system [38]. Moreover, the omnipresent CYP system may be involved in mesalamine oxidation in various tissues and organs, including the kidney, and explains the marked side effects of mesalamine at the level of this organ [2,5,8]. The damaging effects of a reactive mesalamine-derived benzoquinone-imine may be related to the covalently binding protein (ε-amino groups of lysine) and depletion of glutathione with consequent increased oxidative stress, loss of ability of the mitochondria to synthesize ATP and necrosis. Symptoms can be nausea and vomiting, abdominal pain and even acute kidney injury and hepatic necrosis. These aspects are important for clinicians, particularly in relation with the recommended doses of mesalamine.

It was also supposed that 5-ASA (MS) may exert its anti-inflammatory effect in the gut through a proton-coupled electron transfer-based superoxide radical anions (O_2_^•−^) elimination [39]. At the same time, it was found that MS may increase the H_2_O_2_ production [40]. Hydrogen peroxide is a prooxidant factor and it also mediates pro-inflammatory cell-to-cell signaling. Could this be a new therapeutic target for inflammation? Maybe the MS involvement in oxidative processes would necessitate for treatment of intestinal inflammatory diseases, and the association of catalase would enable to degrade excess H_2_O_2_ [41].

## 3. Materials and Methods

### 3.1. Materials

Mesalamine (pharmaceutical grade) was a product of PharmaZell (Raubling, Germany). The laccase, horseradish peroxidase (HRP) and bovine Hb were purchased from Sigma Aldrich (Oakville, ON, Canada). The CP was purified from bovine plasma by chromatography on AE-Agarose, as described by Wang et al. [42]. The Bradford reagent was purchased from BioShop (Burlington, ON, Canada). All other chemicals were reagent-grade and have been used without further purification.

### 3.2. Mesalamine Spontaneous Oxidation—Spectrophotometric Measurements

An aqueous solution of mesalamine (0.55 mM) was stored for up to 6 weeks in order to evaluate spontaneous, catalyst-free oxidation of mesalamine with UV-Vis (200–600 nm) measurements at 1 h, 2 h, 24 h, 2 weeks and 6 weeks. At the end of the interval, infrared (FTIR), ^1^H NMR spectrometry and mass spectrometry were used to confirm mesalamine oxidation. The MS oxidation in presence of H_2_O_2_ was also investigated at room temperature by taking UV-Vis spectra at 1 h, 2 h and 24 h.

### 3.3. Assays of Enzyme Activities

Laccase (EC 1.10.3.2), ceruloplasmin (EC 1.16.3.1), peroxidase (EC 1.11.1.7) are enzymes that belong to class of oxidoreductases, and hemoglobin may act as a peroxidase-like protein. The EC (Enzyme Commission) numbers implemented by the International Union of Biochemistry and Molecular Biology (IUBMB) allow characterization of each enzyme in function of the catalyzed reaction and substrates used. These mentioned enzymes are able to recognize hydroquinone (HQ) as a common substrate and to catalyze its oxidation to *para*-benzoquinone (*p*-BQ) in the presence of oxygen (the oxidases: laccase and ceruloplasmin) or of hydrogen peroxide (peroxidases: peroxidase and hemoglobin). For this investigation, two types of enzymes, oxidases (laccase, CP) and peroxidases (peroxidase and Hb), have each been prepared to present a similar enzyme activity expressed in enzyme units (U) as the amount of enzyme to transform one µmole HQ/min.

The activities of each of the four enzymes have been determined following the method previously described [20]. The rate of increase in absorbance due to enzymatic oxidation of HQ to *p*-benzoquinone (*p*-BQ) was recorded at the fixed wavelength of λ= 250 nm. Briefly, for laccase and CP, the assay medium consisted in 1 mL HQ (0.36 mg/mL) and 1 mL vehicle (50 mM PBS pH 7 or deionized water). Volumes of 0.1 mL laccase or CP enzyme (or vehicle, as control), were added, and the ΔA/min were measured for 5 min. For HRP and Hb the same method was used in similar conditions, with the volume of the assay consisting in 1 mL of HQ (0.36 mg/mL), 1 mL of H_2_O_2_ (0.0163 M) and 0.1 mL HRP or of Hb (or control vehicle: 50 mM PBS pH 7 or deionized water).

The enzyme activities have been calculated using the formula:

$$\mathrm{Enzymatic}\;\mathrm{assay}=\;\frac{∆A/min\times 2.1}{\begin{array}{c} 18.18\times 0.1 \end{array}}$$

where:

2.1 = The total volume the cuvette (in mL)

18.18 = Extinction coefficient of *p*-BQ

0.1 = Sample volume (in mL) used in the assay

Enzyme activity: result expressed in Enzyme Units (U) as μmoles HQ oxidized/min.

### 3.4. Mesalamine Oxidation under Enzyme Catalysis

Various oxidation conditions have been investigated using solutions of mesalamine (MS) 0.55 mM in 50 mM phosphate-buffered saline (PBS) pH 7.

The UV-Vis scans were taken at different time intervals.

#### 3.4.1. The Oxidation of Mesalamine with O_2_-Dependent Copper-Containing Oxidases: Laccase and CP

The MS spectra (200–600 nm) were scanned in 3 mL quartz cuvettes in the absence or in the presence of 0.030 UE of laccase or of CP, in 50 mM PBS pH 7. The total volume of the assay was 2.5 mL: 1 mL MS + 1 mL PBS + 0.5 mL enzyme (at a ratio 2:2:1 for MS: PBS: Laccase or CP) or control/PBS only).

#### 3.4.2. The Oxidation of Mesalamine with H_2_O_2_-Dependent Peroxidase and Peroxidase-like Hb

The UV-Vis spectra were also scanned in 3 mL quartz cuvettes from 200–600 nm for MS in the presence of hydrogen peroxide, with and without HRP or Hb. Briefly, the samples contained 1 mL of MS 0.55 mM, 1.45 mL PBS 50 mM (pH 7), 25 μL H_2_O_2_ (0.05%) and 25 μL of HRP (1.20 mU) or of Hb (1.20mU) or PBS vehicle (control).

### 3.5. Electrochemical Measurements

Mesalamine, acetaminophen, salicylic acid, hydroquinone, and 4-aminophenol were dissolved (10 mM) in 15 mL of a previously de-aerated aqueous electrolyte solution (0.1 M KCl in deionized water). Dissolution was assisted by several minutes of sonication at room temperature. The electrochemical cell was a three-electrode setup: 3 mm diameter standard glassy carbon working electrode (BASi), a standard graphite counter electrode and an Ag/AgCl reference electrode. With an SP-200 potentiostat (Bio-Logic, Seyssinet-Pariset, France), anodic cyclic voltammetry scan windows were optimized to include the oxidation peak of each compound (0 to 0.8 V (vs. Ag/AgCl) for mesalamine, hydroquinone and 4-aminophenol, 0 to 1.0 V (vs. Ag/AgCl) for acetaminophen and 0 to 1.3 V (vs. Ag/AgCl) for salicylic acid. Scan rates were at 100 mV/s. The solutions were tested before and after the addition of the chemicals and each measurement was repeated in duplicate. All measurements were performed in ambient conditions.

### 3.6. Fourier-Transform Infrared (FTIR)

FTIR spectra of the MS and the lyophilized oxidized MS were recorded by a Thermo Scientific Nicolet 6700/Smart iTR (Madison, WI, USA) using a diamond crystal, with 64 scans/min at 4 cm^−1^ resolution in the spectral region of 4000–500 cm^−1^.

### 3.7. ^1^H NMR Spectroscopy

^1^H NMR spectra were recorded on a Bruker spectrometer (300 MHz) (Milton, ON, Canada). All NMR spectra were measured at 25 °C in deuterated solvents. Proton chemical shifts (δ) are reported in ppm. Residual protic solvent DMSO (^1^H, 3.33 ppm).

### 3.8. Mass Spectrometry

High-resolution mass spectra (HRMS) were measured by direct injection of the samples in the Mass Spectrometry Time of Flight (Agilent Technologies, Mississauga, ON, Canada) in electrospray mode by using the analytical platform of the CERMO-FC Center at the University of Quebec at Montreal (UQAM).

## 4. Conclusions

This present study showed that mesalamine, an anti-inflammatory drug of the aminophenol class, undergoes a slow oxidation process that may be accelerated by oxidases with oxygen as a substrate, such as ceruloplasmin, or with hydrogen peroxide as a substrate, such as peroxidases and hemoglobin. The UV-Vis kinetics, FTIR and ^1^H NMR spectroscopy supported the hypothesis of oxidizing mesalamine. Moreover, in the conditions of the present study, an oxidation pathway of mesalamine was suggested as occurring in vivo and the HRMS analysis indicated quinone–imine as a reaction product of mesalamine. The cyclic voltammetry measurements supported the hypothesis of an oxidative process and allowed a ranking of susceptibility to oxidation of mesalamine in comparison with other molecules with related structures showing a higher susceptibility to oxidation of mesalamine, in comparison with Tylenol (acetaminophen) and with Aspirin (salicylic acid).

In conditions of a continuously growing interest in mesalamine as an anti-inflammatory agent, this study contributes to a better knowledge of adverse or even of associated effects of 5-ASA as a drug for treating IBD and other inflammatory dysfunctions.

## Data Availability

Data are contained within the article and Supplementary Materials.

## Supplementary Materials

The following supporting information can be downloaded at: https://www.mdpi.com/1420-3049/28/24/8105/s1, Table S1: Mesalamine oxidation rates with different oxidative enzymes.

## Abbreviations

Au: Absorbance units; CP, ceruloplasmin; FTIR, Fourier transformed infrared spectroscopy; Hb, hemoglobin; ^1^H NMR, proton magnetic resonance; HQ, hydroquinone; HRMS, high resolution mass spectroscopy; HRP, horseradish peroxidase; L, laccase; MS, mesalamine; *p*-BQ, *para*-benzoquinone; PBS, phosphate-buffered saline; U (enzyme units); mU, milli enzyme units; UC, ulcerative colitis.
