# Supplementary material for: Oxidation of Mesalamine under Phenoloxidase- or Peroxidase-like Enzyme Catalysis
Source: Molecules. 2023 Dec 15;28(24):8105. doi: 10.3390/molecules28248105 (PMC10871084; doi:10.3390/molecules28248105)
Supplement: Supplementary file 1 [file molecules-28-08105-s001.zip › molecules-2699957-supplementary.pdf]

molecules

MDPI

Article

Oxidation of Mesalamine under Phenoloxidase- or Peroxidase-like Enzyme Catalysis

Rimaz El Zein[1], Pompilia Ispas-Szabo, Maziar Jafari, Mohamed Siaj and Mircea Alexandru Mateescu [1,*]

[1] Department of Chemistry and Center CERMO-FC, Université du Québec à Montréal, C.P. 8888, Montréal, Québec H3C 3P8

* Author to whom correspondence should be addressed: mateescu.m-alexandru@uqam.ca ; Tel.: (1) 514 987-4319

Table S1 – *Mesalamine oxidation rates with different oxidative enzymes*

Enzymes type	Enzymes units (U) for the oxidation process	Oxidation rates of MS		
**Oxidase (with O_2)**		**ΔA_{230nm}/min**	**ΔA_{330nm}/min**	**ΔA_{460nm}/min**
Laccase	0.030 U	0.00380	0.00291	0.0064
CP	0.030 U	0.00353	0.00244	NA*
**Peroxidase (with H_2O_2)**				
Peroxidase	1.20 mU	0.04760	0.0466	0.09940
Hb	1.20 mU	0.01700	0.0031	0.00064

* NA : Not applicable (Negligible)
